# Attenuation of *Mycoplasma hyopneumoniae* Strain ES-2 and Comparative Genomic Analysis of ES-2 and Its Attenuated Form ES-2L

**DOI:** 10.3389/fvets.2021.696262

**Published:** 2021-06-21

**Authors:** Zhenya Li, Yingxin Wang, Yanyan Zhang, Xibiao Tang, Xiangru Wang, Wenhao Liu, Yulin Qian, Yongwei Zhu, Huanchun Chen, Chen Tan

**Affiliations:** ^1^State Key Laboratory of Agricultural Microbiology, College of Veterinary Medicine, Huazhong Agricultural University, Wuhan, China; ^2^Key Laboratory of Preventive Veterinary Medicine in Hubei Province, The Cooperative Innovation Center for Sustainable Pig Production, Wuhan, China; ^3^Wuhan Keqian Biology Co., Ltd., Wuhan, China; ^4^International Research Center for Animal Disease, Ministry of Science and Technology of the People's Republic of China, Wuhan, China

**Keywords:** *Mycoplasma hyopneumoniae*, virulence, attenuation, *in vitro* passaging, sequence analysis

## Abstract

*Mycoplasma hyopneumoniae* causes swine respiratory disease worldwide. Due to the difficulty of isolating and cultivating *M. hyopneumoniae*, very few attenuated strains have been successfully isolated, which hampers the development of attenuated vaccines. In order to produce an attenuated *M. hyopneumoniae* strain, we used the highly virulent *M. hyopneumoniae* strain ES-2, which was serially passaged *in vitro* 200 times to produce the attenuated strain ES-2L, and its virulence was evidenced to be low in an animal experiment. In order to elucidate the mechanisms underlying virulence attenuation, we performed whole-genome sequencing of both strains and conducted comparative genomic analyses of strain ES-2 and its attenuated form ES-2L. Strain ES-2L showed three large fragment deletion regions including a total of 18 deleted genes, compared with strain ES-2. Analysis of single-nucleotide polymorphisms (SNPs) and indels indicated that 22 dels were located in 19 predicted coding sequences. In addition to these indels, 348 single-nucleotide variations (SNVs) were identified between strains ES-2L and ES-2. These SNVs mapped to 99 genes where they appeared to induce amino acid substitutions and translation stops. The deleted genes and SNVs may be associated with decreased virulence of strain ES-2L. Our work provides a foundation for further examining virulence factors of *M. hyopneumoniae* and for the development of attenuated vaccines.

## Introduction

*Mycoplasma hyopneumoniae* is a pathogen that colonizes the respiratory tract of pigs and can cause porcine enzootic pneumonia (EP), which is an important epidemic disease with very high morbidity and is difficult to eradicate (1–3). Even though mortality rates of EP are low, *M. hyopneumoniae* can colonize and destruct cilia barriers in pigs, which may result in complicated secondary bacterial and viral infections (4, 5). Therefore, it is essential to prevent or control this disease that causes considerable economic losses to the pig industry worldwide (6).

Vaccines play an important role to reduce the prevalence of EP (7, 8). Among them, available inactivated vaccines, based mainly on J strain, do not provide complete protection against lung lesions (9, 10). It has been recently demonstrated that *M. hyopneumoniae* can evade the host's immune protection by surface antigenic variation, inhibiting the complement activation pathway (11, 12). Moreover, more and more studies have revealed that mucosal immunity and cellular immunity play a major role in immune defense against *M. hyopneumoniae* (13, 14). For this reason, attenuated vaccines may thus be a promising alternative (15). Nevertheless, isolating and cultivating *M. hyopneumoniae* is notoriously difficult (16–18); thus, very few attenuated strains have been successfully isolated, which hampers the development of attenuated vaccines (19). Passaging of virulent strains *in vitro* may be a suitable method to produce attenuated strains, and in 2014, Zhang et al. reported an attenuated strain of *Mycoplasma bovis* produced by serial passaging (20). In addition, an attenuated *M. hyopneumoniae* strain 168L has been developed into a commercially available vaccine in China (21), but intrapulmonary injection is required for its usage, which limits its clinical application. In this context, it has enormous significance to develop a new *M. hyopneumoniae* attenuated strain.

Third-generation sequencing has been used increasingly to perform comparative genomics of pathogens (22), and recently, many novel virulence factors and pathogen–host interaction mechanisms of *M. hyopneumoniae* were elucidated using comparative genomics (23). Szczepanek et al. performed comparative genomics of a pathogenic strain of *Mycoplasma gallisepticum* and its attenuated form to identify putative virulence genes (24). However, only a few studies were conducted on the differences between pathogenic *M. hyopneumoniae* strains and attenuated forms using comparative genomics (23).

*Mycoplasma hyopneumoniae* strain ES-2 was isolated and identified and was considered a highly virulent strain through our preliminary work. In order to obtain an attenuated strain of *M. hyopneumoniae*, in the current study, strain ES-2 was serially passaged *in vitro* to produce the attenuated strain ES-2L, and its virulence was evaluated in an animal experiment. More comparative genomics was used to examine differences between the two strains on a genomic level. Our study provides a foundation for excavation of virulence factor and the development of attenuated vaccines against EP.

## Materials and Methods

### *Mycoplasma* Strains and Culture Conditions

*Mycoplasma hyopneumoniae* strain ES-2 (GenBank accession no. CP038641.1) was isolated from EP-affected pigs housed on a farm in Hubei province, China; and its genome was sequenced by State Key Laboratory of Agricultural Microbiology, Huazhong Agricultural University. The pathogen was cultured using modified Friis medium (25, 26) at 37°C in a 5% CO_2_-enriched atmosphere for 48–72 h, until the color of the medium changed from red to yellow (2, 27).

### Virulence Attenuation of Strain ES-2 by *in vitro* Passaging

The strain ES-2 was cultivated in modified Friis liquid medium at 37°C with a 5% CO_2_-enriched atmosphere for 48–72 h and was then passaged to P2. Serial passaging cultivation was carried out as the above way. At passage P197, bacteria were plated on modified Friis solid medium and were cultured in a 5% CO_2_-enriched atmosphere at 37°C for approximately 7–10 days. Individual colony was selected using a low-power microscope and was then cultured in Friis liquid medium. After three cloning and purification steps, the strain produced from the last cloning step (P200) was termed *M. hyopneumoniae* strain ES-2L. During passaging, PCR assays and cell morphology examinations were performed to ensure no abnormality in the passaging process. Genomic DNA of the strain every 40 generations was extracted using a Mycoplasma gDNA Mini Kit (Biomiga, San Diego, CA, USA). The *M. hyopneumoniae-*specific P36 gene and 16SrRNA were amplified using specific primers (Supplementary File 1) with the following PCR program (28, 29): 95°C for 5 min, followed by 28 cycles of 95°C for 30 s, 55°C for 30 s, and 72°C 1 min, 72°C for 10 min, and 16°C for 20 min. Cell morphology was observed by transmission electron microscopy.

### Virulence Evaluation of ES-2 and Passaged Strains

Twenty healthy weaned cross-bred (Landrace × Large Yorkshire) pigs aged 25–28 days were purchased from a local farm in Hubei province, China, which was free of *M. hyopneumoniae* and porcine reproductive and respiratory syndrome virus (PRRSV). Blood samples were collected and were screened for *M. hyopneumoniae* and PRRSV antibodies using an enzyme-linked immunosorbent assay (IDEXX Co., Westbrook, ME, USA). Only animals that are diagnosed with negative antibodies for *M. hyopneumoniae* and PRRSV were used.

Pigs were assigned to five groups of four individuals each, which were designated as ES-2 (P1), P80, P120, ES-2L (P200), and negative control (NC) groups. ES-2 (P1), P80, P120, and ES-2L (P200) pigs were intratracheally injected with ES-2 (P1), P80, P120, and ES-2L (P200) strains, respectively, at 4.9 × 10^8^ color changing unit (CCU). NC pigs were intratracheally injected with an equal volume of sterile Friis medium.

Clinical observations were recorded daily, particularly regarding coughing, which may suggest *M. hyopneumoniae* infection. On days 0, 1, 2, 3, 5, and 7 post-infection, rectal temperature was measured each day before feeding. Pigs were weighed on days 0 and 42 post-infection to calculate average daily weight gain.

On 42 days post-infection, all pigs were anesthetized and then exsanguinated. Lung tissue samples were collected and were fixed in formalin for hematoxylin and eosin (H&E) staining using conventional methods (30) and subjected to PCR assays. Pulmonary lesions were evaluated using the scoring method of Madec and Kobisch (31). Each lung lobe was scored as follows: no pathological lesion, 0 points; 1–25% of the area with lesions, 1 point; 26–50% of the area with lesions, 2 points; 51–75% of the area with lesions, 3 points; and 76–100% of the area with lesion, 4 points (total 28 points, seven lung lobes). For each animal, the lesion score (LS) was calculated from the sum of scores of seven pulmonary lobes and ranged between 0 and 28. This study was conducted after approval by the Animal Experiment Ethics Committee of the State Key Laboratory of Agricultural Microbiology, Huazhong Agricultural University, China (Approval Number: HZAUSW-2019-014). Differences between groups were tested using *t*-tests. Statistical significance is reported at *P* < 0.05.

### Genome Sequencing, Assembly, and Annotation of Strain ES-2

Genomic DNA of *M. hyopneumoniae* strain ES-2L was extracted as indicated above. Then, genomic libraries were sequenced using a combined Illumina NovaSeq (Illumina, San Diego, CA, USA) and Nanopore (Oxford Nanopore, Oxford, UK) sequencing approach (Nextomics, Wuhan, China). Genomes were assembled using Canu software version 1.8 with a combination of short and long reads, followed by error correction using Pilon software version 1.12. A circular chromosome with 2,300-fold average base coverage was produced and then was annotated using the best-placed reference protein set (GeneMarkS+) of the National Center for Biotechnology Information (NCBI) Prokaryotic Genome Annotation Pipeline version 3.3.

### Comparative Genomics and Structure Variation Analysis

For comparative genomic analysis, a genome of *M. hyopneumoniae* primary strain ES-2 was downloaded from GenBank as a reference (accession no. CP038641.1, https://www.ncbi.nlm.nih.gov/nucleotide/CP038641.1). Clean NovaSeq reads were mapped to the reference genome using the Burrows–Wheeler Alignment tool, and structure variations were examined using the three software programs GATK Haplotype Caller version 4.0, FreeBayes version 1.3, and samtools mpileup version 1.8. A genome alignment analysis of strains ES-2 and ES-2L was conducted using the Artemis Comparison Tool.

### Confirmation of Deleted Genes by PCR

Based on the above comparative genomics results, PCR assays were used to confirm whether the predicted genes were really deleted on the genome of strain ES-2. Primers for 18 genes were designed using Primer5 software and were commercially synthesized (TSINGKE Biological Technology, Beijing, China; Supplementary File 1). These genes were amplified by PCR with ES-2 and ES-2L genome templates, using the following program: 95°C for 5 min, followed by 30 cycles of 95°C for 30 s, 55°C for 45 s, and 72°C 1 min, 72°C for 10 min, and 16°C for 15 min.

## Results

### Identification of Strain ES-2L

**Figure 1:**
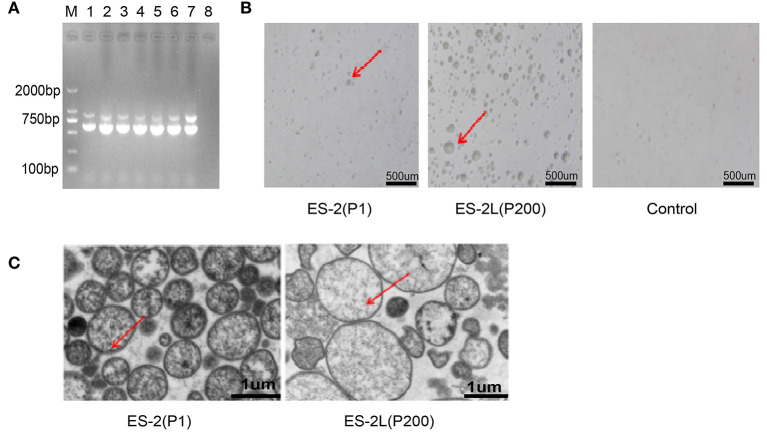
**(A)** Detection of *Mycoplasma hyopneumoniae*-specific P36 (948 bp) and 16S RNA (627 bp) genes for the different passage strains: M, DNA marker 2,000; lane 1, ES-2 (P1); lane 2, P40; lane 3, P80; lane 4, P120; lane 5, P160; lane 6, ES-2L (P200); lane 7, positive control; lane 8, negative control. **(B)** Morphological observation of strain ES-2L and ES-2 on solid medium using a low-power microscope; the colonies of strain ES-2 and ES-2L showed the characteristics of rounded, distinct edges and centers with granules, as indicated by the red arrow. **(C)** Morphological examination of strains ES-2L and ES-2 observed by transmission electron microscopy, indicating that strain ES-2 had no cell walls, cell membranes were surrounded by capsules, and the cytoplasmic structure was loose; characteristics of strain ES-2L were similar to those of strain ES-2, but there were a few large-celled mutant individuals, as indicated by the red arrow.

PCR assays indicated that *M. hyopneumoniae*-specific P36 (948 bp) and 16S RNA (627 bp) target genes could both be detected in ES-2 (P1), P80, P120, P160, and ES-2L (P200) (Figure 1A). Colonies of strain ES-2 and ES-2L on solid medium showed the morphological characteristics of rounded, distinct edges, and centers with granules (Figure 1B), which was accorded with biological characteristics of *mycoplasma*. In addition, transmission electron microscopy displayed that strain ES-2 had no cell walls, cell membranes were surrounded by capsules, and the cytoplasmic structure was loose. The morphological characteristics of strain ES-2L were similar to those of strain ES-2, but there were a few large-celled mutant individuals (Figure 1C).

### Clinical Observations of Virulence Evaluation Experiment

**Figure 2:**
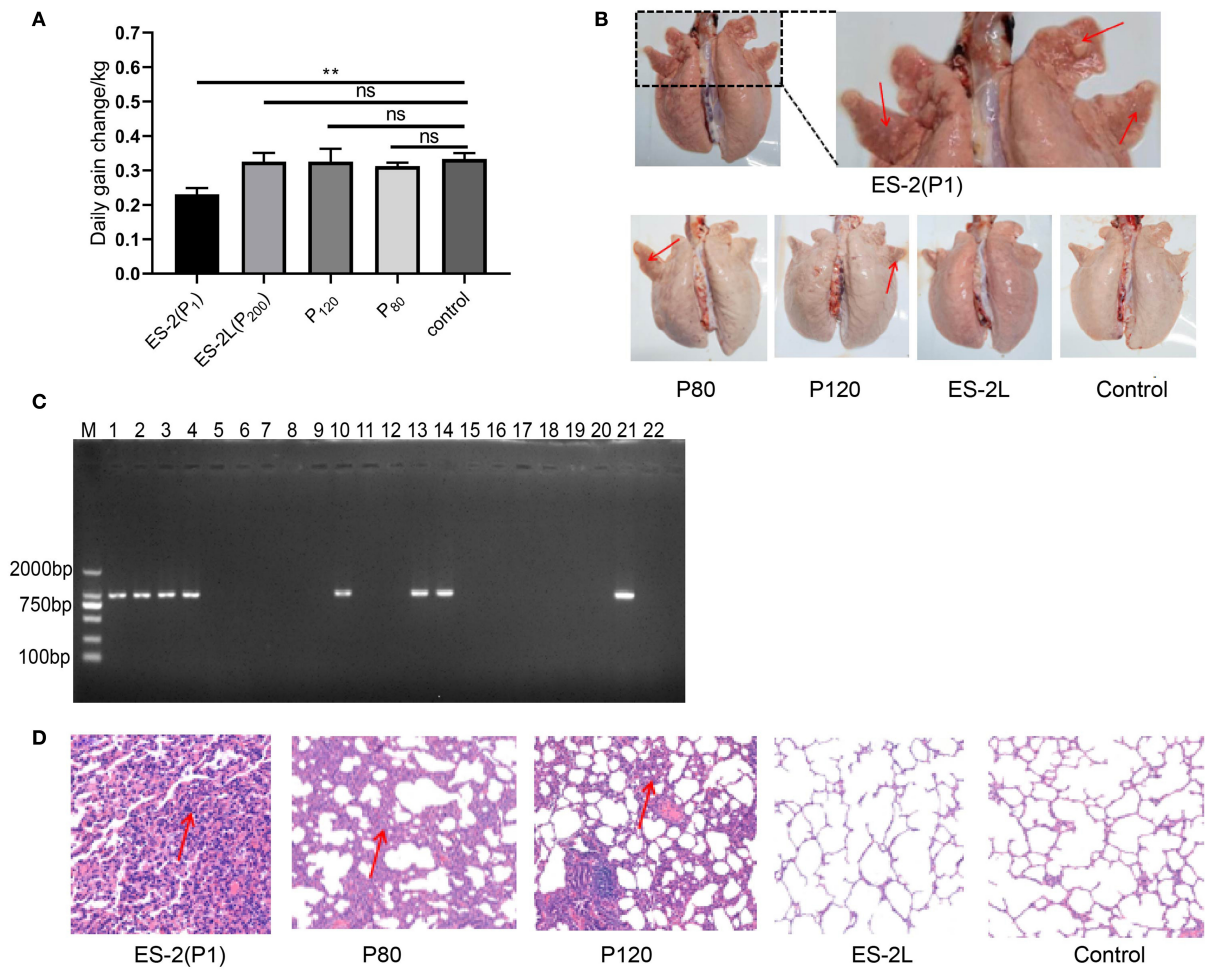
**(A)** Change of average daily weight gain in each group; data are presented as the means ± *SD* (*n* = 4); **P < 0.01, Student's *t*-test. **(B)** Observation of lung tissue macroscopic lesions in each group; all pigs in the P1 group had typical “red consolidations” lesions of lung tissue (indicated by the red arrow). None of the pigs in the ES-2L group showed typical lesions. **(C)** Detection of P36 gene (948 bp) by PCR assay for pig's lungs of each group: M, DNA marker 2,000; lanes 1, 2, 3, and 4 correspond to P36 gene on ES-2 group; lanes 5, 6, 7, and 8 correspond to P36 gene on ES-2L group; lanes 9, 10, 11, and 12 correspond to P36 gene on P120 group; lanes 13, 14, 15, and 16 correspond to P36 gene on P80 group; lanes 17, 18, 19, and 20 correspond to P36 gene on control group; lane 21, positive control; lane 22, negative control. **(D)** Examination of lung tissue hematoxylin and eosin staining by microscopy at 100-fold magnification; the red arrow indicates that there were severe mononuclear cell and lymphocyte infiltration and alveolar consolidation in lung tissue.

No consistent increase in body temperature was recorded in any of the treatment groups after infection. Further, two pigs in the P1 group appeared the symptom of severe cough along with the emaciated and depressed status on day 23 post-infection. Two pigs in the P80 group exhibited moderate cough symptom, and one pig in the P120 group showed mild cough. No coughing was observed in the ES-2L group throughout the experiment period. On the other hand, compared with the NC group, average daily weight gain in the P1 group was significantly reduced (Figure 2A), whereas no difference between the ES-2L group and the NC group was observed. The above results manifested that strain ES-2, not the attenuated strain ES-2L, could induce clinical symptoms of EP in animal infection experiment.

### Macroscopic and Microcosmic Pathological Observations

After dissection on 42 days post-infection, macroscopic lesions were examined. All pigs in the P1 group showed typical “red consolidation” lesions of the lungs (Figure 2B), with the average LS of 11 ± 1. Two pigs in the P80 group had moderate pneumonia lesions in the lungs, with the average LS of 7 ± 2; one pig in the P120 group displayed mild lesions of the lungs, and the average LS was 4 ± 1. None of the pigs in the ES-2L group showed typical lesions in the lungs (Figure 2B), and the average LS was 2 ± 1. In addition, the P36 gene could be detected by PCR assays in all pigs' lungs of the ES-2 group, in two pigs' lungs of the P80 group, in one pig's lungs of the P120 group, and in none of the ES-2L group (Figure 2C). For microcosmic pathological lesions, H&E staining of lung tissue indicated that there were severe mononuclear cell and lymphocyte infiltration and alveolar consolidation in the ES-2 group (Figure 2D). One pig in the P80 group exhibited lesions of severe inflammatory cell infiltration with widened alveolar stroma in the lungs, and one pig in the P120 group had also diseased regions of mild inflammatory cell infiltration in the lungs. No pathological lesions were observed in the ES-2L group (Figure 2D). These results demonstrated that strain ES-2 was virulent, and after serial passaging *in vitro*, its virulence was reduced, resulting in the attenuated strain ES-2L.

### Genomic Characteristics of Strains ES-2L and ES-2

**Figure 3:**
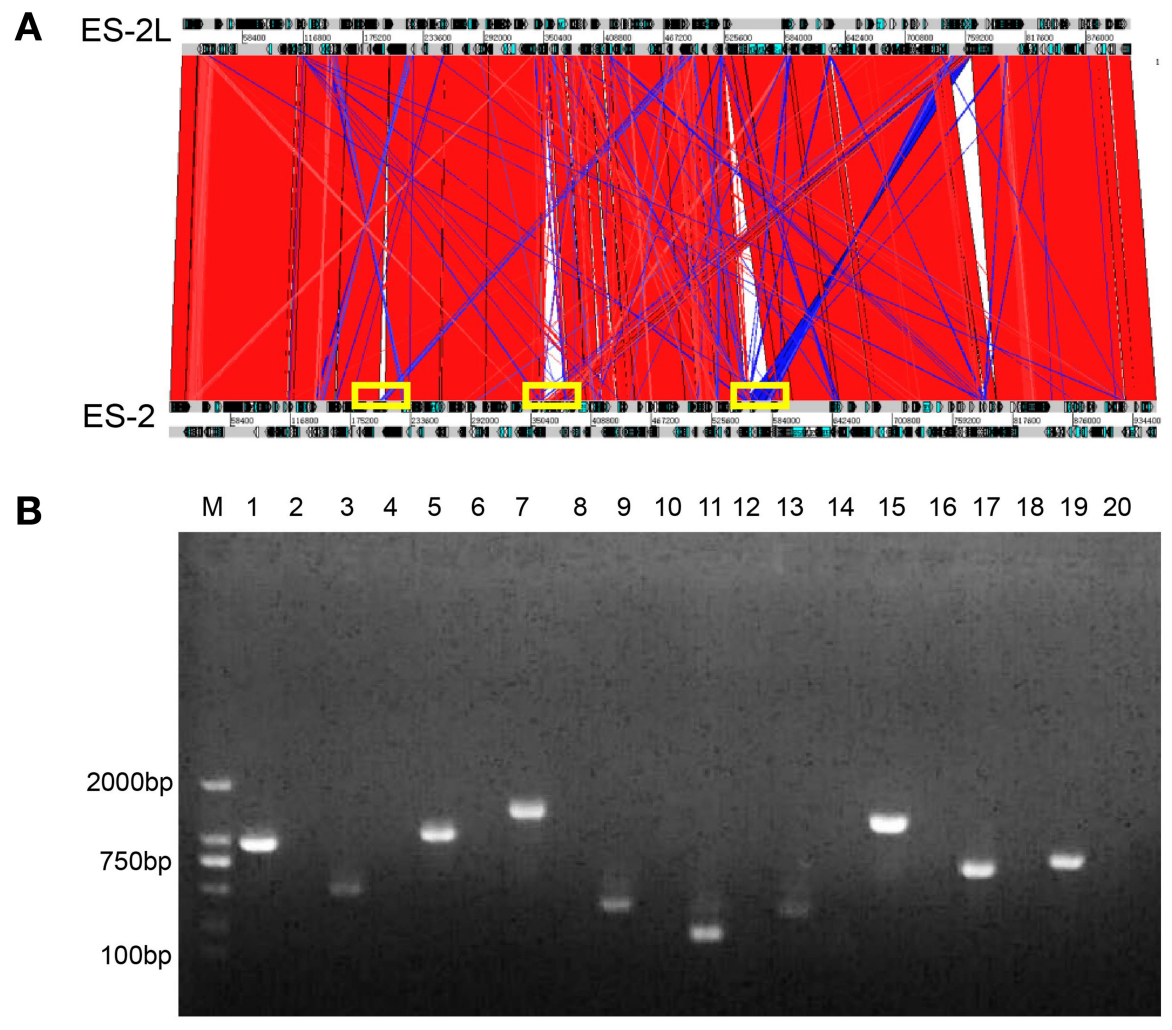
**(A)** Collinearity analysis results of strains ES-2L and ES-2; strain ES-2L showed three large fragment deletion regions indicated by the yellow box, compared with ES-2. **(B)** Confirmation of the deleted genes by PCR. M, DNA marker 2,000; lanes 1, 3, 5, 7, 9, 11, 13, 15, 17, and 19 correspond to genes E5E95_00935, E5E95_00940, E5E95_01845, E5E95_01850, E5E95_01855, E5E95_01860, E5E95_01905, E5E95_03755, E5E95_00670, and E5E95_00665 with strain ES-2 genome as a template, respectively. Lanes 2, 4, 6, 8, 10, 12, 14, 16, 18, and 20 indicate that these genes were amplified with strain ES-2L genome as template.

In total, 108,621 raw reads comprising 2,392,084,536 bases were produced from strain ES-2L (accession no CP058578.1, https://www.ncbi.nlm.nih.gov/nuccore/CP058578.1). Raw reads were filtered and optimized to assemble the first contig with a size of 918,900 bp [guanine–cytosine (GC) content 28.48%]. A total of 697 protein-encoding genes were predicted. The ES-2L genome was 37,614 bp smaller than that of ES-2 (956,514 bp). According to the collinearity analysis results, the ES-2L strain sequence indicated three large fragment deletion regions (Figure 3A), and these large fragment deletion regions indicated 18 gene deletions in ES-2L, compared with ES-2 (Table 1). Analysis of single-nucleotide polymorphism (SNP) and indels indicated that 22 dels were located in 19 predicted coding regions (Supplementary File 2). Except for these indels, 348 single-nucleotide variations (SNVs) were identified between ES-2L and ES-2 (Supplementary File 2). These SNVs mapped to 99 genes where they induce amino acid substitutions and translational stops.

**Table 1:** Eighteen genes in regions deleted from the genome of strain ES-2L.

**Variation type**	**Start site**	**Stop site**	**Size/bp**	**Including gene ID**	**Encoding protein**
Deletion	113,237	114,699	1,462	E5E95_00505	Serine protease
Deletion	159,989	161,778	1,789	E5E95_00670	Hypothetical protein
				E5E95_00665	Hypothetical protein
				E5E95_00675	Hypothetical protein
Deletion	203,386	208,867	5,481	E5E95_00935	Hypothetical protein
				E5E95_00940	Hypothetical protein
Deletion	261,435	262,128	693	E5E95_01210	Hypothetical protein
Deletion	262,717	26,3417	700	E5E95_01215	Hypothetical protein
Deletion	417,189	420,421	3,232	E5E95_01850	Hypothetical protein
				E5E95_01845	Hypothetical protein
				E5E95_01855	Hypothetical protein
				E5E95_01860	Alcohol dehydrogenase
Deletion	429,321	433,928	4,607	E5E95_01905	DNA gyrase subunit A
				E5E95_01910	Hypothetical protein
				E5E95_01915	Serine protease
Deletion	558,155	560,135	1,980	E5E95_02435	IS1634 family transposase
				E5E95_02440	Site-specific DNA-methyltransferase
Deletion	925,798	927,220	1,422	E5E95_03755	DDE domain-containing protein

### Confirmation of Gene Deletion by PCR

Ten of 18 potentially deleted genes, E5E95_00935, E5E95_00940, E5E95_01845, E5E95_01850, E5E95_01855, E5E95_01860, E5E95_01905, E5E95_03755, E5E95_00670, and E5E95_00665, could be detected by PCR, with strain ES-2 genome as template. However, these genes could not be detected when using strain ES-2L genome as PCR template, which indicated that these 10 genes were in fact deleted in strain ES-2L, as confirmed by PCR (Figure 3B).

## Discussion

In this study, the animal infection experiment demonstrated that strains ES-2L and ES-2 have significant difference in virulence. Especially, in terms of clinical symptoms of ES-2 challenged pigs, the ES-2 group developed cough symptoms 23 days after infection; nevertheless, other studies showed that coughing began 14 days after infection (32), which may be caused by the differences of source of the strain, infection dose, and experimental animals. Unlike other pathogens such as PRRSV, *M. hyopneumoniae* could not lead to continuous increase in body temperature after infection (33, 34). In the experiment, regardless of which group, there was no obvious increase in body temperature after infection until the end, which was consistent with other reports (34).

Serum IgG antibody response is not a reliable tool for assessing the infection status of an individual animal (35, 36). For our research, at 28 and 42 days post-infection, the ES-2 and ES-2L groups had a low seroconversion rate (50%), and only one pig in the P120 group was seropositive (results not shown), which could be explained by this mechanism that *M. hyopneumoniae* could weaken humoral immune response by inducing apoptosis of immune cells (37–39). Recently, intensive studies have revealed that IgA antibody level emerges in an uptrend after vaccination, which indicated that mucosal IgA responses play a crucial role in immune protection against *M. hyopneumoniae* (40); hence, it is necessary to detect IgA in the saliva and/or nose after infection in the following investigation.

Pigs infected with virulent *M. hyopneumoniae* strain can have clinical symptoms such as coughing and weight loss, and lung lesions characterized by dark red, firm consolidation in apical, cardiac, and intermediate lobes (6, 41, 42), but further confirmation by molecular diagnosis is still necessary (43). The lungs of healthy piglets infected with strain ES-2 showed typical “well-demarcated, red consolidations” (Figure 2B); especially, *M. hyopneumoniae*-specific gene P36 was detected in the cardiac lobe and apical lobe of the lungs. But gene P36 was not detected by PCR in the spleen, kidney, and liver of all ES-2 challenged pigs, which conflicted with another study that *M. hyopneumoniae* genomes have been detected by qPCR from inner organs, such as the liver, spleen, and kidneys in experimentally infected pigs (30). One possible explanation for the conflict between them was that the detection method adopted on our study was different from theirs. Taken together, these results demonstrated that strain ES-2 was a highly virulent and pathogenic strain, which was also consistent with the results of our previous studies; furthermore, its virulence decreased with the number of *in vitro* passages. Besides, pigs infected with strain ES-2L showed no typical lung lesions and cough symptoms, suggesting the lower virulence of this strain, which may thus be a vaccine candidate. However, further evaluation of its immunogenicity was necessary in the next exploration. Yu et al. obtained also similar macroscopic and microcosmic pathological observations by comparing the virulence of the *M. hyopneumoniae* highly virulent strain 168 and its attenuated strain 168L (32).

In addition, our study found that in the process of continuous passage, the morphology of a small number of cells would undergo the changes to large-celled mutations observed by transmission electron microscopy (Figure 1C). These large-celled mutations contained more DNA compared with typical cell types in the population (44), which might contribute to form biofilms on abiotic surfaces (45), but their role in invading and adhering to host was currently unclear. As expected, the adaptability of strain ES-2L to the culture medium was better than that of strain ES-2, which may be attributed to serial passaging. Specifically, the growth titer of strain ES-2L could reach 1.0 × 10^10^ CCU/ml, which was much higher than that of strain ES-2. Also, the growth rate of strain ES-2L that only took 2 days to grow to the late-log phase was also considerably faster than that of strain ES-2 (results not shown). These phenomena allowed us to hypothesize that some mutated genes related to substance transport and metabolism, such as importing sugars, amino acids, peptides, and metal ions, should be the focus of our attention (46, 47). In line with our assumption, the results of SNP analysis depicted that there were a large number of mutated genes associated with microbial substance transport and metabolism processes, such as ABC transporter protein family.

In order to explain the mechanisms underlying attenuation of strain ES-2, we conducted comparative genomics of ES-2 and ES-2L. The ES-2L genome was 37,614 bp smaller than that of ES-2. During serial passaging, gene deletion may occur, which was observed in *M. bovis* regarding a deletion of a 14.2-kb fragment as assessed by comparative genomics of strain HB0801 and its attenuated form (47). Compared with strain ES-2, strain ES-2L showed several large fragment deletion regions that contained 18 genes (Table 1), and 10 of 18 genes were in fact deleted, as confirmed by PCR (Figure 3B). Unfortunately, the functions of most of these genes are unknown and thus require further examination. One of the deleted genes encodes alcohol dehydrogenase (AdhE; E5E95_01860), which is a key enzyme in the anaerobic fermentation pathway of ethanol and regulates ethanol generation in bacteria (48, 49). Under anaerobic conditions, the lack of AdhE may reduce ethanol synthesis, resulting in decreased hydrogen peroxide concentrations, which was closely associated with the toxicity of mycoplasma (50–52). Interestingly, we further analyzed the genome structure of strain ES-2L and ES-2 by Island Viewer 4 software, which indicated that there are two loss of genomic islands in the process of passaging. Genomic island was considered to be closely related to the horizontal gene transfer (53, 54). According to current researches, the genes encoded by genomic islands were associated with organism virulence, pathogenicity, metabolism, and drug resistance (55, 56). One of the genomic islands, encoding 10 genes, was located at start 920,852-bp site to end 943,294-bp site in the ES-2 genome. Two of the 10 genes, E5E95_03750 and E5E95_03795, encoded LppA family lipoprotein and transcription elongation factor GreA, respectively. Moreover, other researches have reported that LppA family lipoprotein and transcription elongation factor were two important virulence factors of *M. hyopneumoniae*, playing a significant role in the process of *M. hyopneumoniae* invading and adhering to the host (57–60). These findings suggested that the loss of genomic island might be related to the changes in strain ES-2 virulence and growth characteristics.

On the other hand, the results of SNP analysis showed that there were 348 SNVs in strain ES-2L, which may affect the functions of 99 genes. The adherence of *M. hyopneumoniae* to porcine ciliated respiratory cells plays a key step for infection. Liu et al. also exhibited through the comparative genomics results of *M. hyopneumoniae* strain 168 and 168L that there were a large number of SNVs, and these SNVs affected functional expression of some important adhesin gene, such as P97, P102, P46, and P65 (23). E5E95_00610 gene was annotated as adhesin protein by the NCBI Search database; it experienced two non-synonymous gene mutations (C–G and C–T) according to the results of SNP analysis (Supplementary File 2), which suggested that this gene may be a new potential virulence factor, although its biological significance needs further verification. More importantly, *mycoplasmas* can evade the killing of the host's humoral immunity by the MIB–MIP system that captures and cleaves immunoglobulin G (61–63). E5E95_03535 was an important virulence gene encoding putative immunoglobulin-blocking protein, which could bind to the immunoglobulin in the serum to cleave the immunoglobulin, playing a crucial role in evading the neutralization of serum antibodies. The protein undergoes three single-nucleotide mutations (C–T, G–C, and T–C) in the process of continuous passage (Supplementary File 2), which might affect its function. Taken together, gene deletion and SNVs affecting gene function may be responsible for the decrease in virulence of strain ES-2. However, there are still huge technical obstacles to construct gene-deleted strains (64, 65); we are currently unable to study the functions of specific gene through genetic manipulation methods. We look forward to greater progress in related technical operations for verifying the biological functions of these genes.

## Conclusion

We successfully produced an attenuated *M. hyopneumoniae* strain by serial *in vitro* passaging, and lower virulence was confirmed in an animal experiment. Comparative genomics between strains ES-2 and its attenuated form ES-2L indicated numerous considerable differences on a genomic level. Our research provides a new perspective for the pathogenic mechanism of *M. hyopneumoniae* and the development of attenuated vaccines.

## Data Availability Statement

The datasets presented in this study can be found in online repositories. The names of the repository/repositories and accession number(s) can be found below: NCBI GenBank; accession no.'s CP038641.1 and CP058578.1.

## Ethics Statement

The animal study was reviewed and approved by Committee on the Ethics of Animal Experiments at the College of Huazhong Agricultural University.

## Author Contributions

ZL performed experiments, analyses, and wrote the manuscript. ZL performed experiments with the assistance of YQ, XT, WL. YW, and YZhu performed analyses. CT, XW, YZha, and HC designed experiments and revised the manuscript. All authors contributed to the article and approved the submitted version.

## Conflict of Interest

HC and XT were employed by Wuhan Keqian Biology Co., Ltd. The remaining authors declare that the research was conducted in the absence of any commercial or financial relationships that could be construed as a potential conflict of interest.

## Supplementary Material

Supplementary File 1 Primers used for amplification.

Supplementary File 2 Information of single nucleotide polymorphisms (SNP) and indels analysis.
